# Effects of Systemic Physiology on Mapping Resting-State Networks Using Functional Near-Infrared Spectroscopy

**DOI:** 10.3389/fnins.2022.803297

**Published:** 2022-03-08

**Authors:** Androu Abdalmalak, Sergio L. Novi, Karnig Kazazian, Loretta Norton, Tatiana Benaglia, Marat Slessarev, Derek B. Debicki, Keith St. Lawrence, Rickson C. Mesquita, Adrian M. Owen

**Affiliations:** ^1^Department of Physiology and Pharmacology, Western University, London, ON, Canada; ^2^Brain and Mind Institute, Western University, London, ON, Canada; ^3^“Gleb Wataghin” Institute of Physics, University of Campinas, Campinas, Brazil; ^4^Department of Psychology, King’s University College at Western University, London, ON, Canada; ^5^Institute of Mathematics, Statistics and Scientific Computing, University of Campinas, Campinas, Brazil; ^6^Clinical Neurological Sciences, Western University, London, ON, Canada; ^7^Department of Medical Biophysics, Western University, London, ON, Canada; ^8^Department of Psychology, Western University, London, ON, Canada

**Keywords:** functional near-infrared spectroscopy, resting state, systemic physiology, connectivity, short channels

## Abstract

Resting-state functional connectivity (rsFC) has gained popularity mainly due to its simplicity and potential for providing insights into various brain disorders. In this vein, functional near-infrared spectroscopy (fNIRS) is an attractive choice due to its portability, flexibility, and low cost, allowing for bedside imaging of brain function. While promising, fNIRS suffers from non-neural signal contaminations (i.e., systemic physiological noise), which can increase correlation across fNIRS channels, leading to spurious rsFC networks. In the present work, we hypothesized that additional measurements with short channels, heart rate, mean arterial pressure, and end-tidal CO_2_ could provide a better understanding of the effects of systemic physiology on fNIRS-based resting-state networks. To test our hypothesis, we acquired 12 min of resting-state data from 10 healthy participants. Unlike previous studies, we investigated the efficacy of different pre-processing approaches in extracting resting-state networks. Our results are in agreement with previous studies and reinforce the fact that systemic physiology can overestimate rsFC. We expanded on previous work by showing that removal of systemic physiology decreases intra- and inter-subject variability, increasing the ability to detect neural changes in rsFC across groups and over longitudinal studies. Our results show that by removing systemic physiology, fNIRS can reproduce resting-state networks often reported with functional magnetic resonance imaging (fMRI). Finally, the present work details the effects of systemic physiology and outlines how to remove (or at least ameliorate) their contributions to fNIRS signals acquired at rest.

## Introduction

Since the pioneering work of Biswal et al. (1995) with functional magnetic resonance imaging (fMRI), resting-state functional connectivity (rsFC) has gained popularity due to its simplicity and potential for providing insights into various brain disorders (Biswal et al., 1995; Woodward and Cascio, 2015). Typical rsFC studies have simple experimental protocols compared to task-based experiments, which require external stimuli. In rsFC studies, participants are usually instructed to remain still and are asked not to focus their thoughts on any specific task. Although it can be relatively easy for healthy adults to remain still and quiet, this experimental protocol might be challenging for adults with certain brain disorders (e.g., Parkinson’s disease) and for children, particularly infants. After acquiring the data, functional connectivity maps are derived from the neuroimaging signal (e.g., BOLD/ASL in fMRI or hemoglobin concentration changes in fNIRS) by comparing low-frequency spontaneous fluctuations (usually less than 0.15 Hz) from different brain regions (Greicius et al., 2009; Mesquita et al., 2010; Sasai et al., 2011, 2012; Niu et al., 2013; Novi et al., 2016; Tong et al., 2019). These low-frequency oscillations reflect (at some level) spontaneous brain activity and have a systemic physiological contribution. The systemic influences may come from different origins, such as low-frequency spontaneous changes due to breathing, mean arterial pressure, and HR (Battisti-Charbonney et al., 2011; Yücel et al., 2016; Whittaker et al., 2019). Disruptions in rsFC networks have been observed using fMRI in Alzheimer’s disease (Vemuri et al., 2012) and in disorders of consciousness (Abdalmalak et al., 2021), suggesting that patterns of functional brain connectivity during resting state may have potential for diagnosing and monitoring patients with brain disorders.

The most common method of assessing rsFC has been using fMRI. However, other neuroimaging modalities, such as functional near-infrared spectroscopy (fNIRS), have also been used to assess rsFC. fNIRS uses near-infrared light (∼600–900 nm) to continuously and noninvasively measure brain function through temporal changes in oxy- (HbO) and deoxy-hemoglobin (HbR) (Jöbsis, 1977; Ferrari and Quaresima, 2012; Boas et al., 2014; Scholkmann et al., 2014). Due to its high temporal resolution, portability, and affordability, fNIRS is a tool for clinical, basic, and applied research, including continuous monitoring of patients with brain injuries, BCI applications, and neurofeedback protocols (Favilla et al., 2014; Abdalmalak et al., 2017, 2021). In addition, as fMRI and fNIRS signals share similar hemodynamic origins (Huppert et al., 2006a,b; Steinbrink et al., 2006), it is reasonable to hypothesize that fNIRS could, to some extent, reproduce fMRI-based rsFC networks. To that end, Mesquita et al. (2010) recorded fNIRS data from the whole head and found strong inter-hemispheric brain correlations similar to those that are often reported in fMRI studies. In 2011, Zhang et al. (2011) reported that functional connectivity maps derived from fNIRS resting-state data were reproducible at the group level. More recently, graph-theoretical approaches have showed that resting-state fNIRS data is also highly reproducible at the intra-subject level (Niu et al., 2013; Novi et al., 2016). In addition, good agreement has been demonstrated between fMRI and diffuse optical tomography (DOT) in terms of their ability to measure important rsFC networks, such as those involving motor and auditory regions (White et al., 2009; Eggebrecht et al., 2014).

Although promising, fNIRS-based rsFC studies are yet to gain the general acceptance that fMRI-based studies have. Some rs-fNIRS skepticism reflects the fact that the fNIRS signal is highly contaminated by systemic physiology, such as blood pressure changes, breathing, heart rate (HR), and extracerebral hemodynamics (Franceschini et al., 2006; Mesquita et al., 2010; Kirilina et al., 2012; Scholkmann et al., 2013b; Funane et al., 2014; Goodwin et al., 2014; Caldwell et al., 2016; Tachtsidis and Scholkmann, 2016; Yücel et al., 2016; McKetton et al., 2021). In fNIRS experiments, sources and detectors are placed on the scalp, and the diffusively backscattered photons are detected some distance away, bringing hemodynamic information from cortical and extracerebral layers (Saager and Berger, 2005). Since the extracerebral layers are densely vascularized, roughly 94% of the signal measured by a regular fNIRS channel (source-detector distances of ∼3 cm) reflects systemic hemodynamic changes measured from extracerebral tissue, mainly from the scalp (Brigadoi and Cooper, 2015). If not properly removed, these fluctuations can lead to spurious correlations and overestimate functional connections between brain regions (Mesquita et al., 2010). Several preprocessing methods have been proposed to decontaminate the fNIRS signal, such as Global Average Signal Removal, Independent Component Analysis (ICA), and Principal Component Analysis (PCA) (Zhang et al., 2010; Novi et al., 2016; Santosa et al., 2020; Zhou et al., 2021). Among these methods, PCA seems to be the most effective as it does not introduce spurious negative correlations across fNIRS channels (Carbonell et al., 2011). In the PCA framework, one removes the first n-principal components based on the assumption that the extracerebral and systemic physiologies are the highest sources of covariance across channels. One limitation is that the number of components removed is arbitrary and needs further validation.

An alternative approach to removing global systemic physiology (i.e., cortical and extracerebral) from the fNIRS signal is to include short-channel (SC) measurements (Saager and Berger, 2005, 2008; Kirilina et al., 2012; Gagnon et al., 2014; Brigadoi and Cooper, 2015; Yücel et al., 2015, 2021; Pinti et al., 2019; Santosa et al., 2020). SCs refer to fNIRS measurements in which the source-detector distances are on the order of 1 cm or below, with roughly 0.8 cm being the optimum distance for adults (Brigadoi and Cooper, 2015). Due to the small distance between the source and detector, SCs are primarily sensitive to hemodynamics from superficial layers, mainly the scalp (Saager and Berger, 2005, 2008; Brigadoi and Cooper, 2015). Therefore, these signals can later be used as nuisance regressors within the General Linear Model (GLM) framework to decontaminate regular fNIRS channels (Saager and Berger, 2005; Gagnon et al., 2011, 2012; Huppert, 2016; Pinti et al., 2019; Santosa et al., 2020). As SC regressions require additional measurements, which usually comes with the cost of decreasing the number of regular fNIRS channels, fNIRS studies with SC regression have focused on task-based functional protocols. The effects of SC regression on fNIRS-based rsFC maps need to be better understood.

Even though SC regression is an effective method for regressing global systemic noise, several studies have suggested that additional physiological measurements, such as HR, mean arterial pressure (MAP), and end-tidal CO_2_, may provide complementary information that could help reduce false positives and increase the sensitivity to the brain (Scholkmann et al., 2013b; Caldwell et al., 2016; Tachtsidis and Scholkmann, 2016; Nasseri et al., 2018; McKetton et al., 2021). These findings raise further questions about the accuracy of functional connectivity maps recorded with fNIRS even in the presence of SCs.

In the present work, we aimed to investigate the effects of systemic physiology on fNIRS-based rsFC, and to determine the best methods to remove components of the signal associated with systemic variables (i.e., components of the signal not related to cortical activity). We hypothesized that a combination of SCs, HR, MAP, and end-tidal CO_2_ measurements will provide a more precise measurement of the neural and physiological compartments of fNIRS signals acquired at rest, ultimately reducing the intra- and inter-subject variability often observed with fNIRS. To this end, we acquired 12 min of resting-state data from 10 healthy participants. To isolate the contribution of each physiological nuisance signal, we worked within the GLM framework so that we could vary the number of regressors in the model. Finally, we employed PCA to mimic a scenario in which SCs and physiological measurements are not available in order to further validate the use of PCA in fNIRS rsFC studies.

## Materials and Methods

### Experimental Protocol

Data collection was performed at the Brain and Mind Institute at Western University, London, Canada. Ten healthy controls with no history of severe brain injury were recruited (seven females, age range between 22 and 32). Each participant was seated comfortably in a Fowler’s position in a dimly lit room. The average setup time was around 1 h. The experimental protocol consisted of 1 min of baseline (to calibrate the physiological data acquisition systems) followed by 12 min of resting state. The participants were instructed to close their eyes, stay relaxed, and to not focus their thoughts on anything in particular. To ensure that participants did not fall asleep during the study, a research team member monitored the participants’ behavior throughout the entire study. In addition, once the study was completed, participants were asked if they fell asleep at any point during the study. None of the participants reported falling asleep. This study was approved by the Research Ethics Board at Western University, which complies with the guidelines of the Tri-Council Policy Statement (TCPS): Ethical Conduct for Research Involving Humans. All participants provided written informed consent before participating in the study.

### fNIRS Signal Acquisition

**FIGURE 1:**
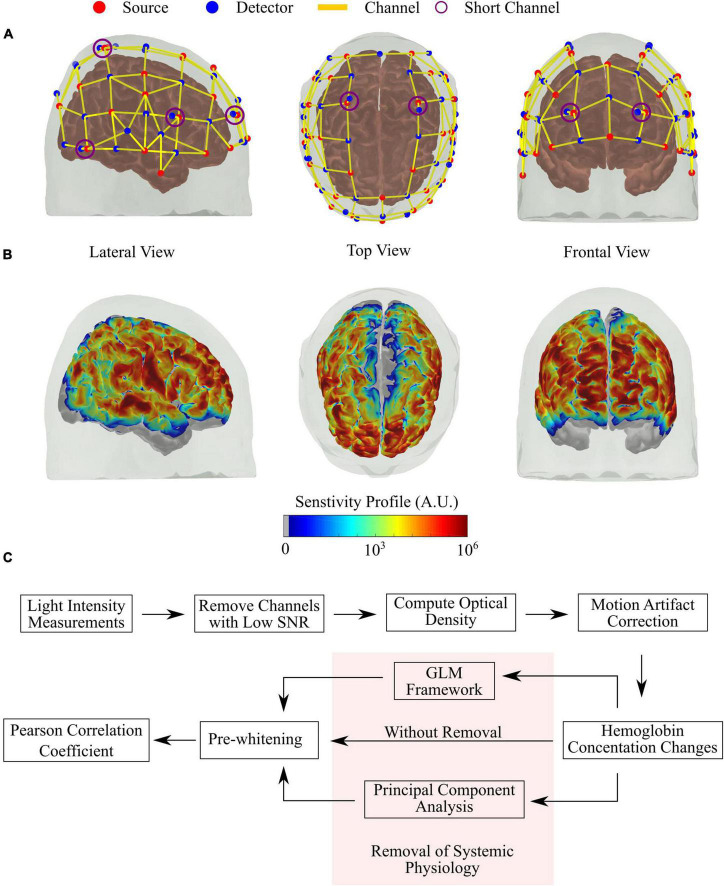
Details of the optical probe and pre-processing steps employed. **(A)** Source-detector configuration of the optical probe used in the study, with 32 sources (red) and 39 detectors (blue), allowing 121 channels (yellow lines) with source-detector separation of around 3 cm and eight short channels (violet circles) with source-detector separation of 0.8 cm. **(B)** Sensitivity profile of the optical probe was obtained with Monte Carlo simulations, considering all channels. **(C)** Flow chart of the pre-processing steps used in this study. The highlighted part is the main focus of this work (see section “Removal of Systemic Physiology”).

We acquired all fNIRS data with a commercial continuous-wave (CW) NIRS system (NIRScout, NIRx Medical Systems) at 3.9 Hz. We designed the optical probe to cover the frontal, parietal, and temporal brain regions with 39 detectors and 32 sources (lasers, centred at 785, 808, 830, and 850 nm), allowing 121 source-detector combinations (i.e., channels) at around 3 cm and eight source-detector pairs (i.e., SCs) at 0.8 cm. The optodes were affixed to the head using a 10–20 standard head cap, presenting a hemispherical symmetry. Figure 1A shows the location of each source and detector on the Colin27 head model available in AtlasViewer, and Figure 1B shows the sensitivity profile of the optical probe generated with Monte Carlo (MC) simulations *via* AtlasViewer (Aasted et al., 2015).

### fNIRS Preprocessing

A summary of the pre-processing steps is shown in Figure 1C. Data analysis were performed using an in-house developed MatLab scripts based on existing Homer 2 functions (Huppert et al., 2009). First, channels with a low signal-to-noise ratio (defined as SNR < 8) were removed. This threshold was based on previous experiences with similar systems (Forero et al., 2017; Novi et al., 2020a). Given the lower number and importance of the SCs in the present work, we also inspected each SC’s power spectrum individually. SCs that did not have a clear peak around the HR frequency (∼1 Hz) and pink noise (1/f decay) were removed from further analysis. Each participant had at least one good short channel that remained in the analysis.

After the quality check, we converted light intensity from the remaining channels to optical density and removed motion artifacts (MA) with a hybrid method that relies on first correcting baseline changes with spline interpolation, then removing spikes by wavelet decomposition (Scholkmann et al., 2010; Molavi and Dumont, 2012; Di Lorenzo et al., 2019; Novi et al., 2020b). The proper removal of MA is crucial in rsFC since they can severely degrade temporal correlations across fNIRS channels. Next, we estimated hemoglobin concentration changes using the modified Beer-Lambert law with a pathlength of six for all four wavelengths. Finally, we band-pass filtered the hemoglobin time series between 0.009 and 0.08 Hz to remove low-frequency drifts and high-frequency physiological noise, such as HR and breathing (White et al., 2009; Mesquita et al., 2010; Sasai et al., 2012; Niu et al., 2013).

### Physiological Recordings and Preprocessing

In addition to the fNIRS data, we simultaneously acquired independent physiological information. HR and MAP were collected with a commercial system (Finapres Medical Systems, Netherlands) whose sensor was affixed to the participants’ left arm to continuously monitor these systemic physiological changes with a sampling rate of 200 Hz. In addition, end-tidal CO_2_ was acquired with a cannula connected to a capnograph (Oxigraph, Inc., United States) with a sampling rate of 4 Hz. Since the Finapres requires calibration before data acquisition, the system was started 1 min prior to the beginning of the experiment. The capnograph was started 30 s prior to the start of the experiment, therefore, the first minute of the Finapres data and the first 30 s of the end-tidal CO2 data were discarded from the analysis. The Finapres and capnograph data were then re-sampled to the fNIRS acquisition frequency (3.9 Hz) and band-pass filtered between 0.009 and 0.08 Hz.

### Removal of Systemic Physiology

We removed systemic physiology from fNIRS signals within the GLM framework (Barker et al., 2013; Kirilina et al., 2013; Huppert, 2016; Yücel et al., 2016; Pinti et al., 2019; Santosa et al., 2020; Wyser et al., 2020). Briefly, each hemoglobin time-series was linearly modelled as:


(1) $${\text{Y}}_{H\,b\,x}=\text{X}\,\beta +\varepsilon ,$$

in which **X** is the design matrix, β is the vector with the model parameters, and ε is the error term (Note, in the equation, *Hbx* = *HbO*,*HbR*). The design matrix contains explanatory variables, such as SC data (**X**_*SC*_), physiological measurements (**X**_*Phys*_), and a constant offset array (**X**_*C*_). To understand the importance of each regressor, we analyzed our data with three GLM models separately: SC regression only (**X**≡[**X**_*C*_,**X**_*SC*_]), systemic physiology only (**X**≡[**X**_*C*_,**X**_*Phys*_]), and both SC and physiology (**X**≡[**X**_*C*_,**X**_*SC* + *Phys*_]), in which **X**_*SC* + *Phys*_≡[**X**_*SC*_,**X**_*Phys*_]. Here, **X**_*SC*_ contained all good SCs (as defined in Section “fNIRS Preprocessing”) to account for heterogeneous scalp hemodynamics, and both HbO and HbR from the SCs were included in the GLM following previous works (Kirilina et al., 2012; Santosa et al., 2020; Wyser et al., 2020). The **X**_*Phys*_ submatrix used MAP, HR, and end-tidal CO_2_ measurements. We allowed a maximum time shift of ±20 s (delaying or advancing each regressor) between each fNIRS channel and each physiological regressor to account for the difference in the transit time between the systemic physiology and the NIRS signal acquired from different brain regions (Tong and Frederick, 2010; Tong et al., 2012). The optimum time lag was defined as the time shift that yielded the highest correlation between the regressor and the regular fNIRS channel. This time shift also accounted for possible synchronization errors between the different acquisition systems (i.e. NIRScout, Finapres and capnograph). In all cases, the filtered signal was written as:


(2) $${\text{Y}}_{f\,i\,l\,t\,e\,r\,e\,d}={\text{Y}}_{H\,b\,x}-\text{X}\,\hat{\beta }$$

after estimating the model parameters ($\hat{\beta }$) that minimize the GLM through robust regression (Barker et al., 2013).

In addition to the GLM regression, we independently investigated the performance of PCA as an alternative approach in the absence of SCs and physiological measurements. The central assumption in PCA neuroimaging analysis is that global physiological noise is the primary source of spatial covariance. Therefore, removing the first principal component of each hemoglobin time series could remove systemic physiological noise (Zhang et al., 2005; Mesquita et al., 2010; Carbonell et al., 2011; Novi et al., 2016; Santosa et al., 2020; Noah et al., 2021). To filter the fNIRS signal with PCA, singular value decomposition was performed on the covariance matrix of the fNIRS signal (${\text{Y}}_{H\,b\,x}^{T}={\text{Y}}_{H\,b\,x}$), and the channel filtering was achieved by subtracting the *n*-first principal spatial components. This procedure was done separately for each chromophore (HbO, HbR). In this work, we focused on removing the first component. We opted to perform SC regression on the hemoglobin time series for two main reasons. First, since the SC regression aims to remove systemic physiology, we prefer to perform it on physiological data, which is the hemoglobin time series in the case of fNIRS. Second, SC regression on the hemoglobin time series facilitates the comparison of SC regression with the other procedures (additional physiological measurements and PCA) to remove systemic physiology (Santosa et al., 2020; Noah et al., 2021).

### Correlation Analysis and Seed-Based rsFC Networks

We employed pre-whitening in the filtered HbO and HbR concentration time series *via* an autoregressive model to remove temporal autocorrelation from the fNIRS signal (Barker et al., 2013; Santosa et al., 2017a), reducing spurious correlations across the brain (Arbabshirani et al., 2014; Santosa et al., 2017a; Blanco et al., 2018). After this procedure, total hemoglobin concentration (HbT) was estimated as the sum of HbO and HbR, and the Pearson correlation coefficient across the regular fNIRS channels was computed. We investigated how the different regression approaches affected the correlation distributions (upper diagonal of the correlation matrix) for each participant and hemoglobin. We performed group analysis *via* the concatenation of the correlation distributions of each participant.

In addition to the correlation distributions, we extracted the most common rsFC networks that have cortical contributions (sensorimotor, auditory, FPC, and DMN networks) with the previously validated seed-based method (Biswal et al., 1995; Beckmann et al., 2005; De Luca et al., 2005, 2006; Damoiseaux et al., 2006; Van den Heuvel and Hulshoff Pol, 2010; Van Dijk et al., 2010; Eggebrecht et al., 2014). To do so, we converted the correlation coefficients to Z-scores *via* Fisher’s transformation then we averaged the Z-score coefficients across participants to compute an average connectivity matrix per hemoglobin. Finally, we selected one seed (i.e., regular channel) per network then back-projected its Z-scores to the brain. The chosen seeds were located at the left precentral gyrus over the primary motor cortex (sensorimotor network), left superior temporal gyrus (auditory network), left middle frontal gyrus (FPC network), and right superior frontal gyrus (DMN network). To visualize the seed-based networks, we used the sensitivity profile of each channel computed through MC simulations (see Section “fNIRS Signal Acquisition”) to project to the cortex the correlation value of that specific channel with the chosen seed.

### Inter- and Intra-Subject Variability Analysis

Finally, we investigated the effect of systemic physiology on the variability of the extracted seed-based rsFC networks. To do so, we quantified the variability at the inter-subject level by computing the Euclidian distance between the seed-based maps from a pair of participants across all possible combinations (in total, 45 combinations per network per preprocessing). We also investigated the stability of the rsFC networks over time by dividing the original 12-min fNIRS time-series into two 6-min segments, extracting the rsFC networks for each segment and then calculating the Euclidian distance between each network with the network found when using the entire time series. We also computed the Euclidian distances between the networks extracted with each segment.

## Results

### Systemic Physiology Inflates Correlation Distributions

**FIGURE 2:**
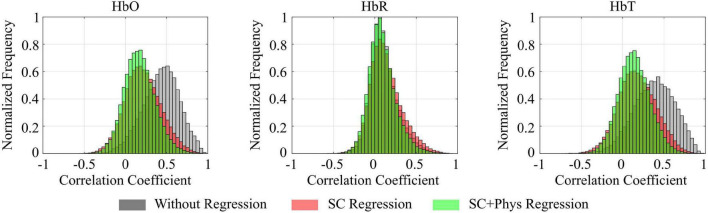
Combined correlation distributions for all participants calculated for oxy-hemoglobin (HbO), deoxy-hemoglobin (HbR), and total hemoglobin (HbT) concentration time series for different preprocessing approaches: before any regression is performed (gray), after regressing only the short channels (SC, red), and after regressing the short channels and systemic physiology (SC+Phys, green). Extracerebral regression has a large impact on the removal of spurious correlations.

Figure 2 shows the correlation distributions for HbO, HbR and HbT for all participants when the signals were not regressed (gray), after regressing the SCs only (red), and after regressing SCs and systemic physiology (green). The regressions shifted the HbO and HbT distributions toward smaller values, indicating that a large component of the observed correlations in standard fNIRS signals was due to extracerebral hemodynamics rather than spontaneous brain fluctuations (false positives). More specifically, the distribution mode *(*i.e., the most frequent correlation coefficient) decreased from 0.51 (without regression) to 0.18 (after SC regression) for HbO and from 0.42 to 0.14 for HbT. The inclusion of the additional physiology in the GLM model did not change the HbO and HbT distribution modes but decreased the spread of the correlation coefficients by removing additional covariance not accounted for by the SC regression. In addition, we observed that the mode of HbO and HbT both shifted to 0.22 when only the physiological regressors were used in the GLM (data not shown), suggesting that SCs contain more information than just the one embedded in HR, MAP, and end-tidal CO_2_. In fact, although SCs and independent physiological measurements account for most of the extracerebral hemodynamic fluctuations, they provided complementary information that could not be obtained by either regressor separately. The extracerebral oscillations do not significantly affect correlations performed with HbR; the distribution mode remained at 0.06 for all approaches, even after the regressions were performed.

**FIGURE 3:**
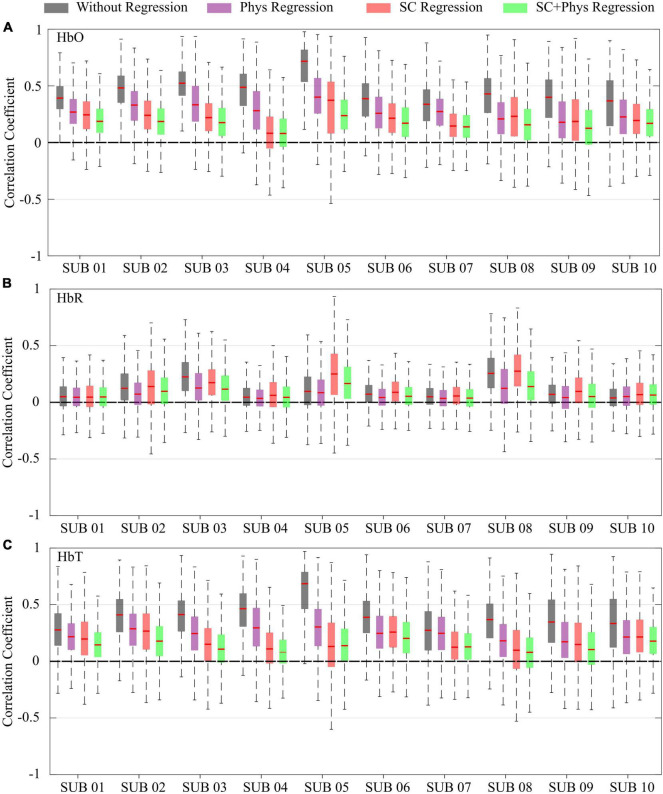
Box plot for the correlation coefficients for each participant and preprocessing method for **(A)** oxy-hemoglobin (HbO), **(B)** deoxy-hemoglobin (HbR), and **(C)** total-hemoglobin (HbT) concentrations. The different colors correspond to different preprocessing approaches: no extracerebral regression (gray), after regression with additional physiological measurements (violet), after regression with short channels (red), and after regression with short channels combined with physiological measurements (green).

The pattern observed for the group was consistent across all participants. Figure 3 shows how the different regression approaches affect the correlation coefficients for each participant and chromophore. Again, one can observe that the correlation values decrease as the number of explanatory regressors in the GLM is increased for HbO and HbT. The combined SC + physiology regression is the most effective approach to remove spurious correlations in all participants. However, when both are not available, regression with SC is preferred over independent physiology since the former removes more spurious correlations than the latter alone in most cases. For HbR, the effect of each regression is not as evident and reproducible as for HbO and HbT. In fact, for most participants (eight out of 10), visual inspection suggests that the regression does not affect the HbR correlation distributions.

### Removal of Systemic Physiology Localizes Functional Connectivity Maps

**FIGURE 4:**
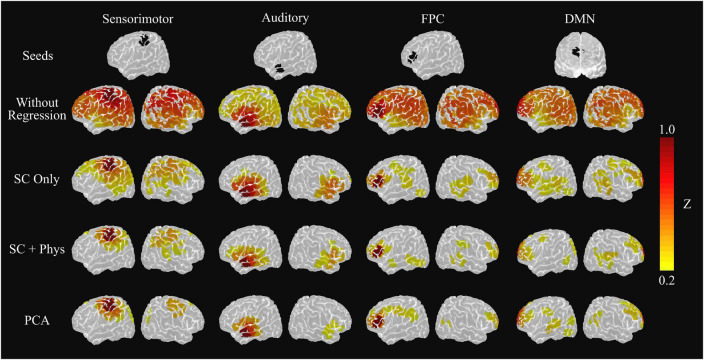
Group level sensorimotor, auditory, frontoparietal control (FPC) and default mode network (DMN) seed-based networks extracted from the average HbT correlation matrix. The top row shows the location of the seed used to extract each map. The following rows represent the resulting networks after using a given method to remove systemic physiology, including PCA regression after removing the first principal component. The removal of systemic physiology better localizes the networks and increases the agreement with the fMRI literature.

The decrease in the number of meaningful coefficients impacts the rsFC networks built with the standard seed-based approach. Figure 4 shows the group average rsFC networks when using the HbT time series for the different processing approaches. (Note, we opted to focus on HbT since it accounts for both HbO and HbR, and previous work has shown that HbT provides higher reproducibility in comparison to the two chromophores separately (Sheth, 2004; Culver et al., 2005; Novi et al., 2016). However, for completeness, the average maps obtained using HbO and HbR are presented in Supplementary Figures 1,2, respectively.) Using the fNIRS signals without removing systemic physiology leads to a highly correlated brain, even after motion artifact correction and pre-whitening were employed. One can observe that the chosen seeds correlate with most brain regions covered by our montage, suggesting systemic components in the fNIRS signal that result in large spatial covariance. However, when the systemic physiology is removed, most irrelevant brain connections are eliminated, and only the expected connections for each network remain. The decrease of spurious correlations is more evident as the number of regressors in the GLM increases, better localizing the rsFC networks. For the sensorimotor and auditory networks, only inter-hemispheric connections with contralateral-homotopic brain regions remain after regression, similar to those previously reported in fMRI. Interestingly, the PCA approach provided similar group sensorimotor and auditory networks to those after SC + systemic physiology regression.

For the cognitive rsFC networks [i.e., the cortical regions of the default mode network (DMN) and the frontoparietal control network (FPC)], the SC regression was not enough to remove all spurious correlations. In fact, the chosen seeds still yielded significant connections with other brain regions that are not typically considered part of the DMN/FPC networks, such as the primary and secondary motor cortices. On the other hand, the SC + physiology regression provided more localized networks in agreement with previous fMRI studies. In this latter case, the channel from which the FPC network was extracted shows correlations with the frontoparietal (left hemisphere) and frontotemporal connections, while the channel from which the DMN was extracted reveals connections among the left pre-frontal cortex, the left posterior parietal lobe, and the posterior temporal gyrus from both hemispheres. Although the rsFC networks with PCA are similar to the SC + physiology regression, the PCA-based FPC network lacks connections with the temporal lobe.

**FIGURE 5:**
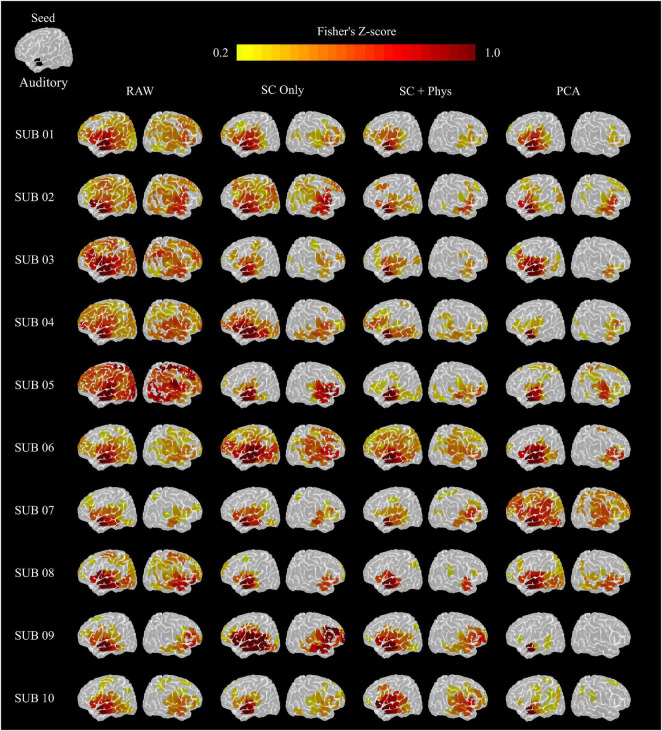
Auditory networks extracted from the HbT correlation matrix for each participant. The seed was located in the left superior temporal gyro (top left image). The removal of systemic physiology better localized the rsFC networks in most participants.

The group results were consistent at the single-subject level. Figure 5 shows the single-subject auditory networks extracted using the seed-based approach from the HbT correlation matrices. As in the group results, the removal of systemic physiology led to better localization in most participants. The auditory network showed significant correlations in the left and right temporal lobes after SC + physiology regression in all participants, and the remaining spurious correlations were notably observed for only two participants (4 and 6). Surprisingly, PCA removed spurious correlations in most cases (eight out of the ten participants) while maintaining correlations in the main areas commonly found in the auditory networks. It is worth noting the PCA performance, given the simplicity of the method that does not require additional measurements. The other rsFC networks investigated showed the same behavior (see Supplementary Figures 3–5).

### Removal of Systemic Physiology Decreases Inter- and Intra- Subject Variability

**FIGURE 6:**
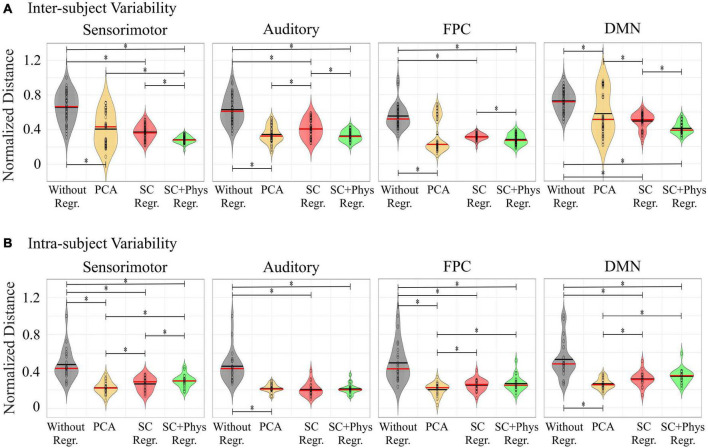
Violin plots with **(A)** inter- and **(B)** intra-subject variability estimated through the Euclidian distance among networks from pairs of participants. The red and black lines inside each violin are the median and mean values, respectively. The black circles are actual values. To verify the impact of each preprocessing approach on the inter- and intra-subject variability, we normalized the distances by the maximum values across all preprocessing approaches within each network. (*) denotes pairs of variability distributions statistically different (*p* < 0.05) from each other assessed with a two-sided *t*-test.

Visual inspection of Figure 5 suggests that removal of systemic physiology not only localizes the rsFC networks but also improves reproducibility across participants. We quantified the variability at the inter-subject level by calculating the Euclidian distance between a pair of rsFC networks considering all possible combinations (normalized by the maximum values across all preprocessing within each network) as described in Section “Inter- and Intra-subject Variability Analysis” (Figure 6A). The removal of systemic physiology significantly decreased the average inter-subject variability in all networks (*p* < 0.05, two-sided *t*-test). The SC and SC + physiology approaches yielded rsFC networks across participants with the highest precision, as noted by their significantly lower interquartile range from 0.29 to 0.57 and 0.24 to 0.46, respectively (Table 1). Even though PCA could be used to extract rsFC networks without any systemic physiological information, the inter-subject variability with this approach was not precise, with an interquartile range from 0.08 to 0.89. The high dispersion of the inter-subject variability in the rsFC networks after PCA regression is likely related to the amount of covariance that can be accounted for in the first component. In our data, the first component accounted for covariances ranging from 24 to 64% and 16 to 55% across all participants for HbO and HbR, respectively (PCA is applied on HbO and HbR prior to computing HbT; therefore, no covariance is reported for HbT). Overall, the decrease in variability after removing systemic physiology strongly suggests that physiological noise must be accounted for before comparing results across participants; otherwise, differences between participants could be due to their systemic physiology and may have not originated from actual neural sources.

**TABLE 1:** Average (interquartile range) Euclidean distance across different pairs of participants.

	rsFC networks			
	Motor	Auditory	FPC	DMN
No physiological regression	0.66 (0.53–0.81)	0.63 (0.51–0.75)	0.55 (0.45–0.62)	0.73 (0.62–0.82)
SC only	0.37 (0.32–0.43)	0.41 (0.30–0.50)	0.31 (0.29–0.34)	0.49 (0.46–0.57)
SC + physiology	0.28 (0.26–0.31)	0.33 (0.28–0.37)	0.28 (0.24–0.34)	0.41 (0.34–0.46)
PCA	0.41 (0.08–0.60)	0.34 (0.28–0.41)	0.34 (0.19–0.56)	0.58 (0.40–0.89)

Finally, we also explored how regressing extracerebral physiology affects the intra-subject variability in fNIRS rsFC. To do so, we divided the original 12-min HbT time series into two 6-min segments. Figure 6B shows the normalized distances (i.e., the distances divided by the maximum values across all preprocessing approaches within each network) for the FPC, DMN, sensorimotor and auditory networks. The removal of systemic physiology also decreased the intra-subject variability for all networks. In this case, PCA had the highest performance on decreasing intra-subject variability, while the SC + physiology regression did not improve the intra-subject reproducibility compared to the SC-only regression. In addition to comparing the first and second halves of the time series to the entire time series, we also estimated the variability in the maps between the first and second segments and observed the same pattern as a function of the different preprocessing approaches (results not shown). These findings suggest that physiological regression leads to more stable rsFC networks even when shorter acquisition times are considered. However, we acknowledge that one would not expect large changes in the spatial-temporal features of the systemic physiological noise during 12 min of resting state. For fNIRS data acquired during different sessions, these features can change, thus decreasing the performance of PCA, while other methods utilizing additional measurements could account for these unpredictable changes.

## Discussion

The purpose of the current study was to evaluate the effects of systemic physiological variables on fNIRS rsFC by acquiring resting-state data with SCs and additional physiological measurements. Our results are in agreement with previous studies and reinforce the fact that systemic physiology could result in false positives, thus overestimating rsFC (Mesquita et al., 2010; Kirilina et al., 2012; Tong et al., 2012, 2013; Scholkmann et al., 2014; Caldwell et al., 2016; Tachtsidis and Scholkmann, 2016; Yücel et al., 2016, 2021; Santosa et al., 2017b,^2020^; Wyser et al., 2020). These contaminations increase intra- and inter-subject variability, which decreases the ability to detect changes in rsFC and may mislead group comparisons or even longitudinal single-subject interpretations. Unlike previous studies, we investigated the efficacy of different pre-processing approaches and how the application of these different techniques affects the reproducibility of rsFC networks. Interestingly, although SC regression reduces the contribution of extracerebral hemodynamics, the remaining signal still contains systemic information associated with independent physiological data. We also observed that fNIRS could extract resting-state networks often reported with fMRI only if systemic physiology is removed, and when the appropriate channels are chosen as seeds.

The results shown in Figures 2, 3 reveal that HbO and HbT are more heavily contaminated by extracerebral physiology than HbR. There is a clear decrease in correlation post-SC and SC + physiology regression for HbO and HbT, whereas this trend is absent for HbR. The low sensitivity of HbR to systemic physiology concurs with the results of previous studies and is likely related to the arterioles being drained to a greater extent than the venules during the activity of the autonomic nervous system, thus affecting the HbO signals to a larger degree than the HbR signals (Franceschini et al., 2003; Tachtsidis and Scholkmann, 2016; Sutoko et al., 2019). On the other hand, the lower signal-to-noise ratio (SNR) of HbR cannot be ignored as it will decrease the contribution of the actual neural hemodynamic fluctuations compared to noise, disrupting the power to detect rsFC networks from HbR at the group level, as can be seen in Supplementary Figure 2. A larger cohort will likely be needed to find significant changes in HbR due to its low SNR. In this study, we focused our analysis on HbT based on previous work (Sheth, 2004; Culver et al., 2005; Novi et al., 2016). However, results using HbO were similar to those obtained with HbT (Supplementary Figure 1).

We also investigated whether PCA could be an adequate alternative when SCs or additional physiological measurements are unavailable. To our knowledge, this is the first rsFC study that validates PCA with SC regression. PCA is attractive as it is simple to implement and does not require additional measurements (such as SCs). The efficacy of PCA relies on the fact that global physiology is the primary source of spatial covariance across fNIRS channels. Therefore, removing the first *n* principal components of the fNIRS covariance can eliminate systemic physiology from the data. The major drawback of the technique is that the number of components to remove is arbitrary. Regressing a large number of components can lower the sensitivity to actual hemodynamic fluctuations of neural origin (introducing false negatives), while removing too few may not be enough to remove all systemic physiology (introducing false positives). To ameliorate this problem, the covariance (usually 80%) to be regressed is often defined, rather than the number of components (Franceschini et al., 2006; Santosa et al., 2020). In the present work, we additionally investigated the effect of removing two components or defining 80% of the covariance to be regressed. Overall, our results suggest that removing the first component yielded the best agreement with SC and SC + physiological regression. We observed that removing the first two principal components (average covariance of 62 and 45% for HbO and HbR, respectively) produced rsFC networks with no inter-hemispherical connections. In contrast, incrementing the number of components until reaching at least 80% of covariance resulted in removing most of the correlation across channels, producing meaningless networks. Particularly for our dataset, this procedure removed on average 6 and 10 components for HbO and HbR, respectively. Overall, our results indicate that it is advisable to remove only the first principal component for our particular dataset.

An important application of rsFC is the ability to extract reproducible functional networks associated with normal brain function. The introduction of physiological regression approaches increases inter- and intra-subject reproducibility (Figure 6). At the inter-subject level, SC + physiology regression provided rsFC networks with the lowest variability, suggesting that MAP, CO_2_ and HR do provide complementary information for SC measurements, which is in agreement with previous studies (Scholkmann et al., 2013a,b; Caldwell et al., 2016). Although PCA had the highest inter-subject variability, it significantly decreased the variability compared to the data without regression. Note that the observed inter-subject variability with PCA was likely from participants 07 and 08. For these specific participants, regressing only one component was insufficient for localizing the rsFC networks (see Figure 5).

For the intra-subject analysis, we observed a similar pattern, i.e., regression of physiological measurements is crucial to extract stable fNIRS-based rsFC networks. Interestingly, PCA had the highest performance on decreasing intra-subject variability. This is likely because we truncated the time series into two segments, and therefore the contribution of systemic physiology was the same for both runs. The efficacy of PCA could vary for studies where data is acquired on different days since the contribution of systemic physiology may not be consistent between sessions. Unlike PCA, independent measurements will always account for varying systemic physiology between days. Another result is that SC + physiology regression did not improve the intra-subject reproducibility compared to the SC-only regression. This finding suggests that SC regression may be sufficient for longitudinal studies focused on the intra-subject level. Further analysis with data acquisition performed over different days and different times within the same day are necessary (Novi et al., 2020a). Nevertheless, our results show that systemic physiology needs to be removed (i.e., whether with SC regression, additional systemic physiological regression or PCA) for longitudinal studies to provide meaningful results.

A seed-based approach was used to extract rsFC networks in this work as this approach has proven reliable in this context (White et al., 2009; Eggebrecht et al., 2014). A challenge, however, is choosing the appropriate seed location for each network, particularly for standard fNIRS systems. For this study, the location of the seeds was chosen based on previous studies (Eggebrecht et al., 2014). To investigate the effect of alternate seed locations on the extracted networks, we computed the rsFC networks for all possible seeds (Supplementary Video 1) for the SC + physiology regression method. Overall, the highest correlations occurred for inter-hemispheric connections with contralateral-homotopic brain regions for HbO, HbR, and HbT in most seeds. One approach to overcoming the challenge of choosing a particular seed is to use independent component analysis (ICA) to extract rsFC networks (Khan et al., 2021). For fNIRS, this approach has been shown to provide reproducible sensorimotor rsFC networks (Zhang et al., 2011) and has been extensively validated with fMRI (Smith et al., 2009). Therefore, it appears to be a promising alternative to seed-based analysis using fNIRS.

Although dozens of rsFC networks have been reported in the fMRI literature, we were primarily interested in four specific networks (sensorimotor, auditory, FPC, and DMN, Figure 4), chosen for two main reasons. First, these networks are known to be robust, being often reported across different populations and neuroimaging techniques (Biswal et al., 1995; Beckmann et al., 2005; De Luca et al., 2005, 2006; Damoiseaux et al., 2006; Van den Heuvel and Hulshoff Pol, 2010; Van Dijk et al., 2010; Eggebrecht et al., 2014). Second and more importantly, the investigated networks have high clinical relevance, providing important information about brain health. In particular, the DMN and FPC play a critical role in normal consciousness (Demertzi et al., 2014; Stender et al., 2014). Previous work has shown that disruption in these networks could be a potential marker for patients who are likely to suffer from a disorder of consciousness (Demertzi et al., 2014; Stender et al., 2014; Kazazian et al., 2021). Therefore, extracting these networks at the single-subject level illustrates the potential of fNIRS as a tool for assessing awareness at the bedside, longitudinally and with low cost.

An important methodological consideration when regressing MAP, HR, and end-tidal CO_2_ is the transit time of blood circulation, which will introduce a temporal shift between the physiological signals acquired in peripheral vasculature and the fNIRS measurements on the head. Previous work has shown that low-frequency oscillations (such as those observed in the MAP signals) measured in the periphery (i.e., finger and toe) are strongly correlated to the rsFC measured with the BOLD contrast in fMRI with varying time delays (Tong et al., 2013). The low-frequency oscillations travel with the blood, and the delay observed is due to the difference in arrival times reflecting the vessel size, pathlength and flow rate of the blood to different sites (for example, the finger). As these oscillations can arrive to the brain earlier or later than the periphery (with respect to the measurement sampling), we allowed a maximum shift of ±20 s to improve the regression’s performance. We observed a higher removal of spurious correlations when we allowed this shift compared to zero-lag regressions.

While the present work is a good step toward resolving physiological artifacts in fNIRS signals so that it might be used in a similar fashion as fMRI in the context of diagnosis of brain health, it also has some notable limitations. First, the sample size was limited to 10 participants. We believe that although a larger sample size could increase the statistical power of our findings, the observed reproducible effect of removing systemic physiology across participants is reassuring and indicates that the observed results were not random. Moreover, the decrease in inter- and intra- subject variability after regressing physiology also increases the statistical validity of our findings. Second, the number of useable SCs varied across participants. More specifically, the number of participants and good SCs (# of participants, # of good SCs) were: (3, 1); (1, 2); (2, 4); (3, 5); and (1, 8). The different number of SCs per participant could potentially affect the performance of the SC regression due to non-homogenous spatial-temporal features of extracerebral hemodynamics across the head (Gagnon et al., 2011, 2012; Kirilina et al., 2012). Although one may argue that a better SC performance could potentially eliminate the need for acquiring MAP, HR and CO_2_, we observed that regressing these physiological parameters removed spurious correlations not accounted for by SCs. To further evaluate the limitation of the number of available SCs, we repeated the group analysis by only including participants that had at least three good SCs. We obtained similar results (figures not shown) as the ones shown in Figures 2, 4. Therefore, we believe that additional physiological measurements do provide complementary information to extracerebral hemodynamics recorded using SCs. Finally, the fNIRS cap was positioned on the head using a standard procedure based on the 10–20 system. This could lead to variability in cap placement across participants, thus affecting the sensitivity of the probes to underlying brain areas Abdalmalak et al. (2020). A more sophisticated approach using a real-time neuronavigation system could decrease the variability in cap placement and increase the average correlation within each brain network, further strengthening our results (Novi et al., 2020a; Wu et al., 2021).

## Conclusion

In conclusion, this work investigated the effects of systemic physiology on fNIRS-based rsFC networks. Our main results agree with previous studies and reinforce the fact that systemic physiology results in spurious correlations, misleading results and conclusions. Our work expanded on previous work by investigating different pre-processing approaches, including SC regression, SC + physiology regression, and PCA. Our results suggest that the best approaches for reducing the effects of systemic physiology and decontaminating the fNIRS signals are (in this order): combination of SC and additional physiological measurements, SC only, and finally PCA. All these approaches showed good performance for extracting meaningful rsFC networks (such as those observed with fMRI). Overall, the present work opens new directions to investigate emerging applications with fNIRS, such as monitoring functional connectivity in patients with acute brain injury at the bedside.

## Data Availability Statement

The data supporting the findings of this research are available on request to the corresponding author (AA), pending a formal data sharing agreement and approval from the local ethics committee.

## Ethics Statement

This study was approved by the Research Ethics Board at Western University, which complies with the guidelines of the Tri-Council Policy Statement (TCPS): Ethical Conduct for Research Involving Humans. All participants provided written informed consent before participating in the study.

## Author Contributions

AA, SN, RM, and AO contributed to the conception and design of the study. AO, KL, and MS provided the equipment necessary for data collection. AA and KK collected the data for the study. SN with inputs from AA and RM carried out the data analysis. AA and SN wrote the manuscript with inputs from all the authors. LN and TB provided feedback on the methods and results to ensure scientific rigor. AO and RM secured funding for the study. All authors contributed to critically revising the manuscript for important intellectual content and approved the final version of the manuscript for publication.

## Conflict of Interest

The authors declare that the research was conducted in the absence of any commercial or financial relationships that could be construed as a potential conflict of interest.

## Publisher’s Note

All claims expressed in this article are solely those of the authors and do not necessarily represent those of their affiliated organizations, or those of the publisher, the editors and the reviewers. Any product that may be evaluated in this article, or claim that may be made by its manufacturer, is not guaranteed or endorsed by the publisher.
