# Supplementary material for: Effects of Systemic Physiology on Mapping Resting-State Networks Using Functional Near-Infrared Spectroscopy
Source: Front Neurosci. 2022 Mar 8;16:803297. doi: 10.3389/fnins.2022.803297 (PMC8957952; doi:10.3389/fnins.2022.803297)
Supplement: Supplementary file 1 [file Data_Sheet_1.DOCX]

Supplementary Material


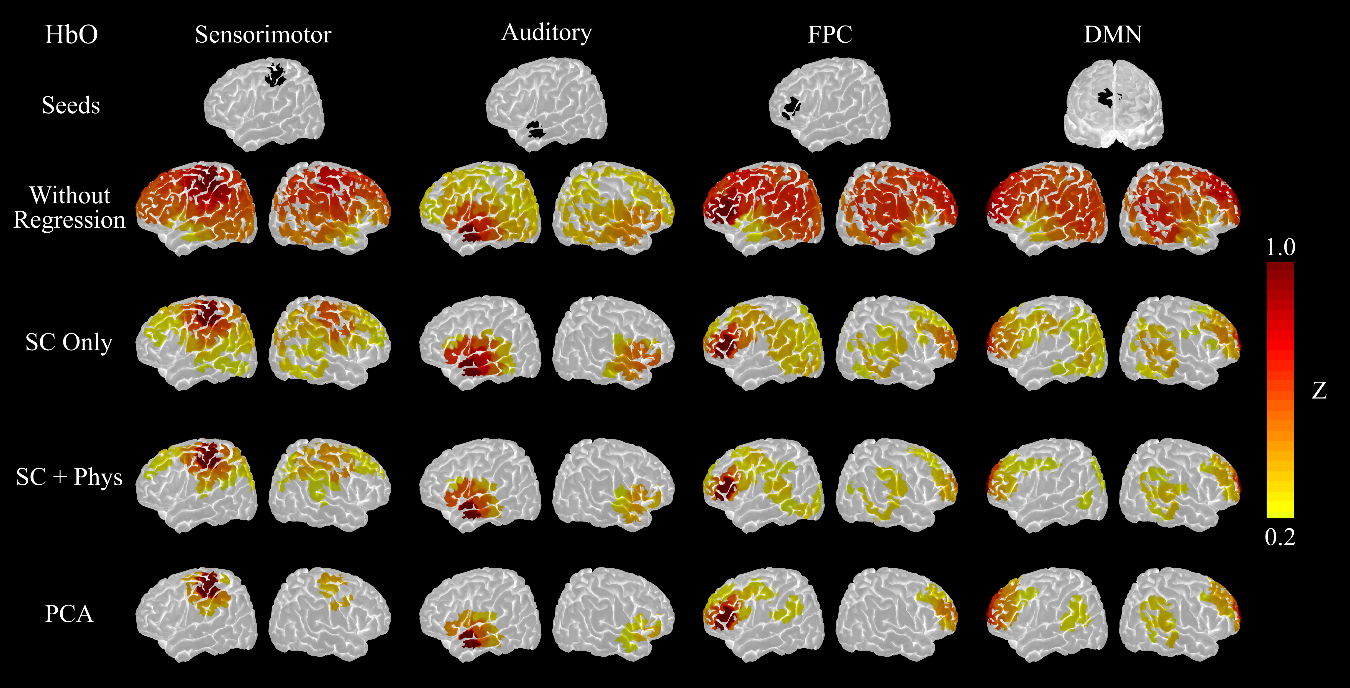


Supplementary Figure 1: Sensorimotor, Auditory, frontoparietal control (FPC) and default mode network (DMN) seed-based networks extracted from the average HbO correlation matrix. First line shows the location of each seed used to extract each map. Each row represents the resulting network after using a given method to remove systemic physiology, including PCA with removing the first principal component. The removal of systemic physiology localizes the networks, increasing the agreement with the fMRI literature.


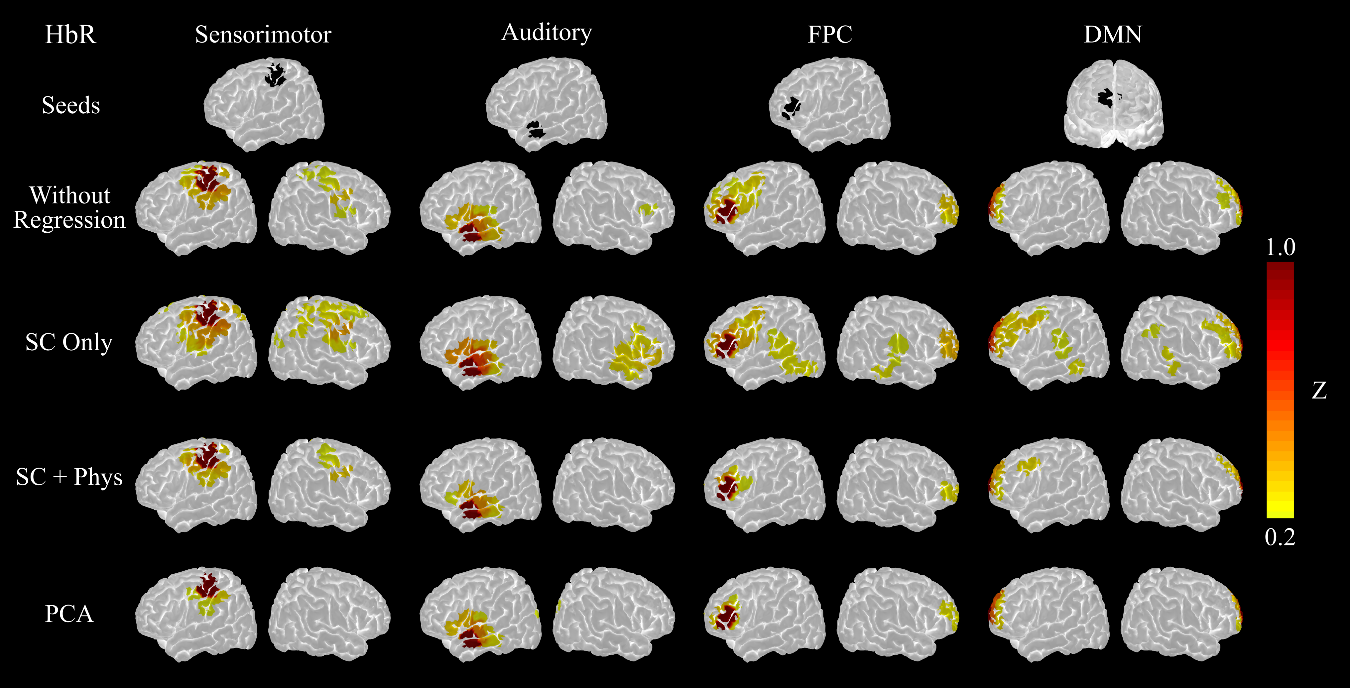


Supplementary Figure 2: Sensorimotor, Auditory, frontoparietal control (FPC) and default mode network (DMN) seed-based networks extracted from the average HbR correlation matrix. First line shows the location of each seed used to extract each map. Each row represents the resulting network after using a given method to remove systemic physiology, including PCA with removing the first principal component. The removal of systemic physiology localizes the networks, increasing the agreement with the fMRI literature.


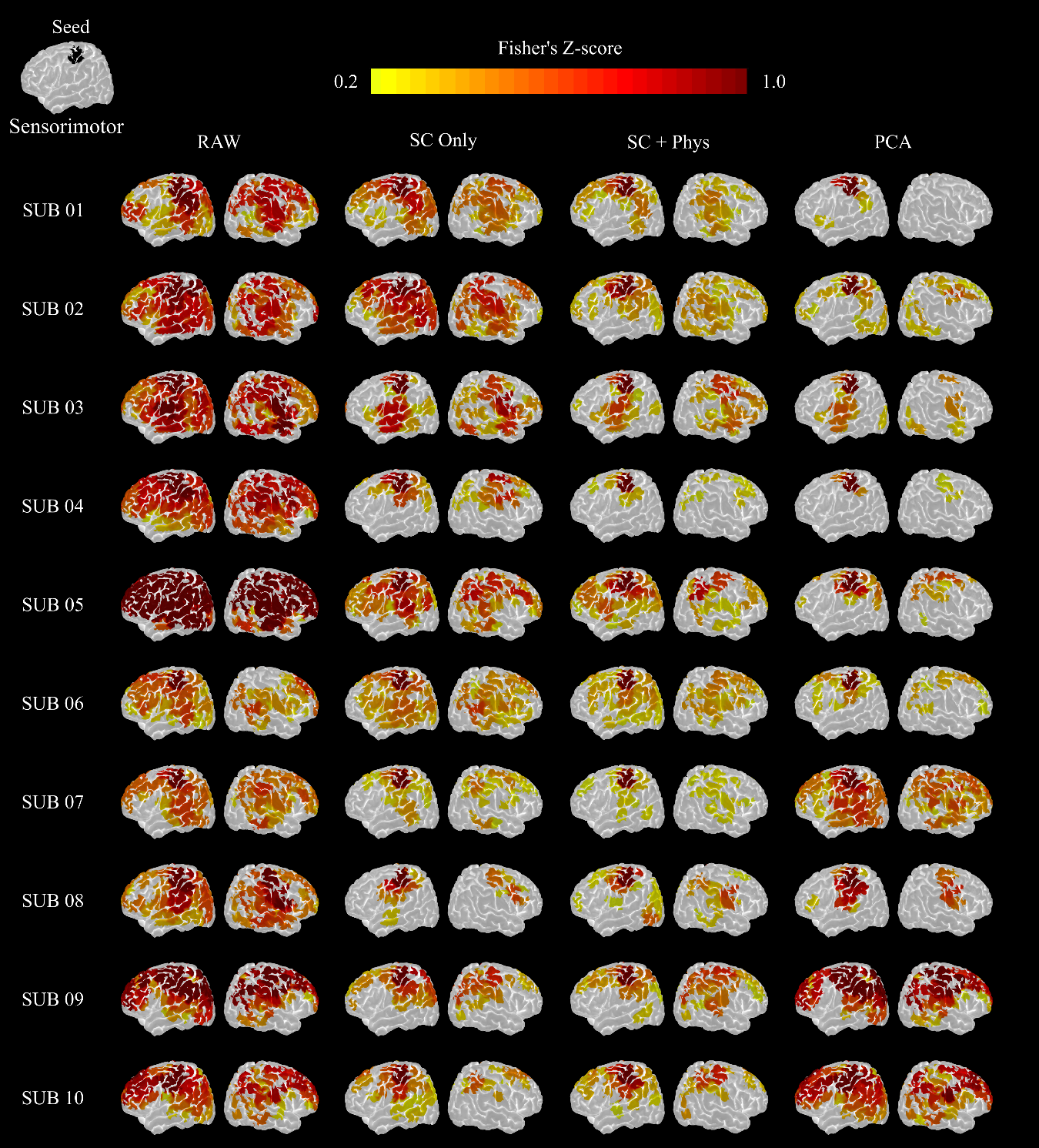


Supplementary Figure 3: Sensorimotor seed-based networks extracted from HbT correlation matrix from each volunteer. The seed is located in the left primary motor cortex (see left top image). The seed location is the same as the one used in Figure 4. The removal of systemic physiology localizes the seed-based map for each volunteer. For PCA, we removed only the first principal component.


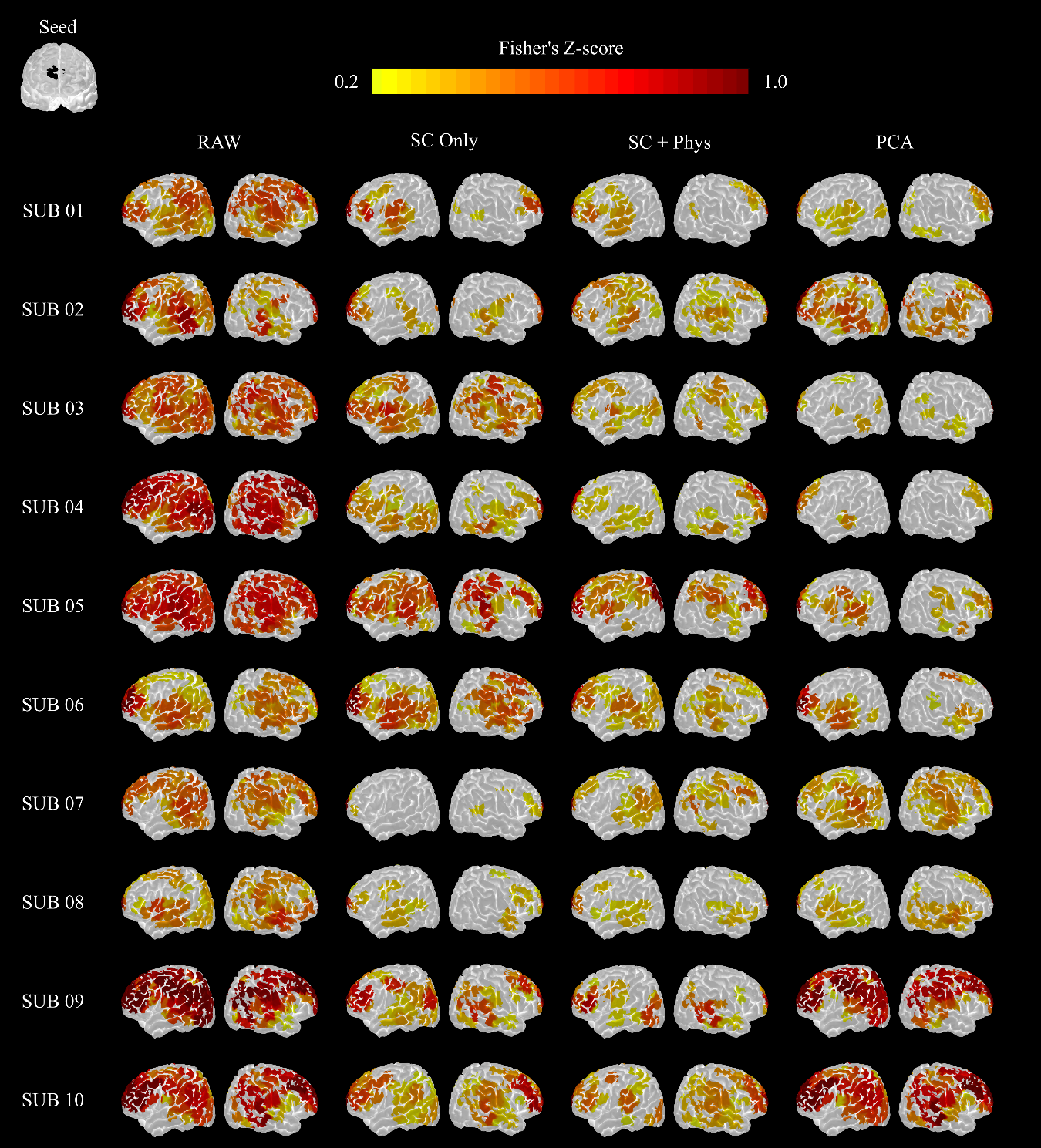


Supplementary Figure 4: Default-mode network seed-based networks extracted from HbT correlation matrix from each volunteer. The seed is located in the right frontal cortex (see left top image). The seed location is the same as the one used in Figure 4. The removal of systemic physiology localizes the seed-based map for each volunteer. For PCA, we removed only the first principal component.


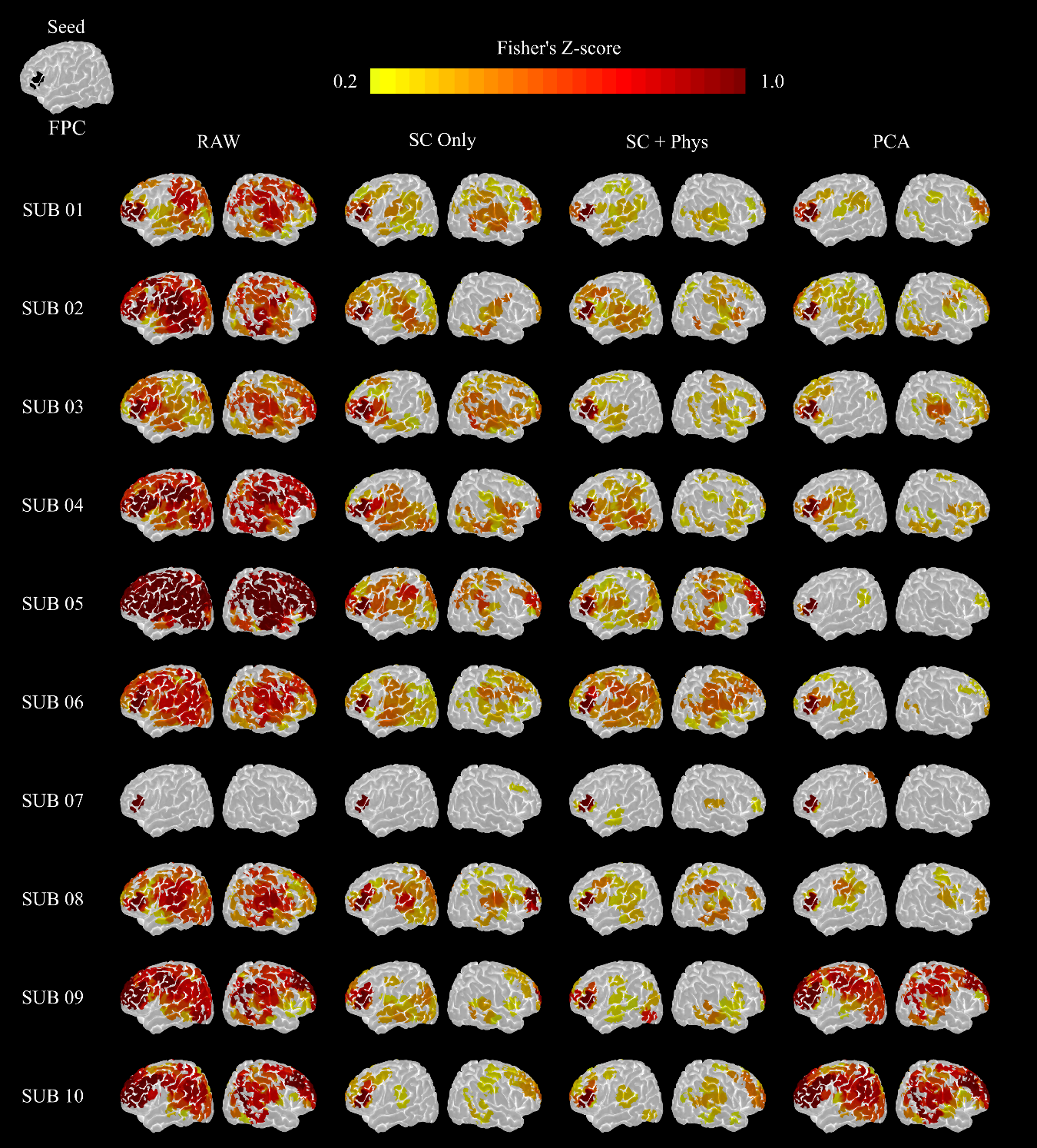


Supplementary Figure 5: Frontoparietal control (FPC) seed-based networks extracted from HbT correlation matrix from each volunteer. The seed is located in the left frontal cortex (see left top image). The seed location is the same as the one used in Figure 4. The removal of systemic physiology localizes the seed-based map for each volunteer. For PCA, we removed only the first principal component.

**Supplementary Video 1:** [rsFC for all possible seed-based networks](https://www.youtube.com/watch?v=Lf7QKRK8woo).
