# Supplementary material for: Effects of Systemic Physiology on Mapping Resting-State Networks Using Functional Near-Infrared Spectroscopy
Source: Front Neurosci. 2022 Mar 8;16:803297. doi: 10.3389/fnins.2022.803297 (PMC8957952; doi:10.3389/fnins.2022.803297)
Supplement: Supplementary file 2 [file Table_1.DOCX]

Table 1. Average (interquartile range) Euclidean distance across different pairs of subjects.

|  | rsFC networks | | | |
| --- | --- | --- | --- | --- |
|  | Motor | Auditory | FPC | DMN |
| No physiological regression | 0.66 (0.53 – 0.81) | 0.63 (0.51 – 0.75) | 0.55 (0.45 – 0.62) | 0.73 (0.62 – 0.82) |
| SC only | 0.37 (0.32 – 0.43) | 0.41 (0.30 – 0.50) | 0.31 (0.29 – 0.34) | 0.49 (0.46 – 0.57) |
| SC + physiology | 0.28 (0.26 – 0.31) | 0.33 (0.28 – 0.37) | 0.28 (0.24 – 0.34) | 0.41 (0.34 – 0.46) |
| PCA | 0.41 (0.08 – 0.60) | 0.34 (0.28 – 0.41) | 0.34 (0.19 – 0.56) | 0.58 (0.40 – 0.89) |
